# A method for developing regulatory gene set networks to characterize complex biological systems

**DOI:** 10.1186/1471-2164-16-S11-S4

**Published:** 2015-11-10

**Authors:** Chayaporn Suphavilai, Liugen Zhu, Jake Y Chen

**Affiliations:** 1 Purdue University, School of Science, Department of Computer & Information Science, Indianapolis, IN, USA; 2 Indiana University School of Informatics and Computing, Indianapolis, IN, USA; 3 Institute of Biopharmaceutical Informatics and Technology, Wenzhou Medical University, Wenzhou, Zhejiang Province, China

**Keywords:** Gene set networks, gene regulation, regulatory gene set networks, pathway crosstalk

## Abstract

**Background:**

Traditional approaches to studying molecular networks are based on linking genes or proteins. Higher-level networks linking gene sets or pathways have been proposed recently. Several types of gene set networks have been used to study complex molecular networks such as co-membership gene set networks (M-GSNs) and co-enrichment gene set networks (E-GSNs). Gene set networks are useful for studying biological mechanism of diseases and drug perturbations.

**Results:**

In this study, we proposed a new approach for constructing directed, regulatory gene set networks (R-GSNs) to reveal novel relationships among gene sets or pathways. We collected several gene set collections and high-quality gene regulation data in order to construct R-GSNs in a comparative study with co-membership gene set networks (M-GSNs). We described a method for constructing both global and disease-specific R-GSNs and determining their significance. To demonstrate the potential applications to disease biology studies, we constructed and analysed an R-GSN specifically built for Alzheimer's disease.

**Conclusions:**

R-GSNs can provide new biological insights complementary to those derived at the protein regulatory network level or M-GSNs. When integrated properly to functional genomics data, R-GSNs can help enable future research on systems biology and translational bioinformatics.

## Background

Researchers often use pathway analysis [1] to reveal novel insights into their gene lists obtained from high-throughput experiments. One simple approach is evaluating the number of genes from a differentially expressed gene list found in a particular pathway. Advanced methods calculate pathway-level statistics. In some studies, a pathway is also considered to be a gene set, a group of genes sharing common biological functions. For example, Gene Set Enrichment Analysis (GSEA) calculates gene set-level statistics [2]. Using gene set-level statistics has additional advantages because significant analysis at the single gene level suffers from a limited number of samples and noise [3]. Gene set based methods have also been developed to investigate phenotypic changes at the pathway level [4]. By using pathway topology, researchers can obtain a better ranking of pathways/gene sets from their gene lists [1].

Several approaches have been proposed for constructing networks of pathways/gene sets to study complex molecular networks. Yong, et al. constructed a global pathway crosstalk network and linkage network for yeast [5]. Dikla, et al. proposed a method for gleaning patterns of interactions among biological processes by analyzing protein-protein interactions, transcriptional co-expressions, and genetic interactions [6]. Gene set networks can be also applied to understanding diseases. Liu, et al. proposed an approach to detect the crosstalk among Alzheimer's disease (AD) related pathways and the dysfunctions in the six brain regions of AD patients [7]. For databases of pathway/gene set network, Sudhir, et al. developed Human Pathway Database (HPD) to enable the study of human pathway networks [8]. Huang, et al. developed Pathway And Gene-set Enrichment Database (PAGED) to further study human gene set networks [9]. Another study proposed an approach to examine interactions between pathways in mice by integrating different types of data [10]. Recently, Jignesh, et al. proposed methods to construct multi-edge gene set networks (GSNs) to reveal insights into global relationships among biological themes or gene sets [11]. A multi-edge GSN consists of three types of edges: co-membership edge (M), linkage edge (L), and co-enrichment edge (E).

To our knowledge, none of the existing gene set or pathway networks contain high-level directionality information, which is essential for building models to understand the mechanistic linkages among many gene sets or pathways that are enriched in a biological condition. In this paper, we present a method of constructing a directed, regulatory gene set network called regulatory gene set network (R-GSN). A directed edge in an R-GSN represents a regulatory relationship from one gene set to another. Compared to a linkage gene set network (L-GSN), which is constructed from protein interactions discarding directionality of the interactions, an R-GSN provides higher resolution of knowledge. Our hypothesis is that R-GSNs can reveal novel gene set relationships and provide complementary knowledge to the existing types of GSNs, such as M-GSNs, L-GSNs, and E-GSNs.

In an R-GSN, a pair of gene sets are connected if a significant number of gene regulations exist between the unique genes of the gene sets. Gene set and gene regulation data were collected from multiple sources. To evaluate R-GSNs, M-GSNs are constructed separately as baselines for each collection including KEGG, Reactome, and three types of Gene Ontology (GO) terms. We exploited the directionality information provided by R-GSNs to search for significant gene sets in the network. We also compared our R-GSNs with E-GSNs obtained from Jignesh et al [11]. We chose Alzheimer's disease (AD) as an example to study disease-specific R-GSNs. In order to construct an AD specific R-GSN, gene sets from different collections were first combined into one single collection and then AD related gene sets were used to construct the AD specific R-GSN.

## Methods

In a gene set network (GSN), a node represents a gene set and an edge represents a relationship between two gene sets. A GSN helps explain biological complexity by revealing high level relationships among biological processes. We constructed a new type of GSN, a regulatory gene set network (R-GSN), using public available gene regulation data. Another type of GSN we constructed is the co-membership gene set network (M-GSN), which can be a baseline network as it is constructed from annotated gene sets and thus provides experimental validation. Finally, we used hypergeometric distribution to calculate significance values for each edge in both R-GSNs and M-GSNs.

### Data sources

The two major types of data used to construct R-GSNs are gene set data and gene regulation data. Gene set data provides information for nodes in a gene set network as well as information for constructing co-membership gene set network. Five collections of gene sets were collected. They included KEGG, Reactome, GO Biological Process, GO Cell Component, and GO Molecular Function from KEGG [12] and MSigDB [2]. The total number of gene sets from the five gene set collections is 2,304 and the total number of genes is 11,111.

Gene regulation data was used for constructing R-GSNs. Human gene regulations from String [13], TRANSFAC [14], TRED [15], and SPIKE [16] were collected. Different types of ID of genes obtained from different data sources were mapped to NCBI official gene symbols. In order to select only the high quality human gene regulation data, different criteria were used to filter gene regulations in different data sources. For String, gene regulations with score >= 800 were collected; for TRANSFAC, binding site quality <= 5 was the criterion; and for TRED, only gene regulations not obtained from computational predicted method were collected. All gene regulations provided by SPIKE were collected since they are from pathway. The total count of unique gene regulations after combining data from the four data sources is 22,127.

### Construction of gene set networks

An M-GSN was constructed separately for each gene set collection. The hypergeometric distribution was used to calculate the significance value for each co-membership edge.

1. Count the number of genes inside each of the two gene sets, GS1 and GS2, and the number of shared genes between GS1 and GS2.

2. Calculate the p-value by using the hypergeometric distribution:

(1) $$p-value=\left(\begin{array}{c} \text{K}\\ \text{k} \end{array}\right)\left(\begin{array}{c} \text{N}-\text{K}\\ \text{n}-\text{k} \end{array}\right)/\left(\begin{array}{c} \text{N}\\ \text{n} \end{array}\right)$$

where N is the total number of genes; n is the number of genes in GS1; K is the number of genes in GS2; and k is the number of shared genes.

3. Adjust the p-value for multiple hypotheses to control the false discovery rate by using the Benjamini-Hochberg procedure with p-value ≤ 0.05.

4. Connect a pair of gene sets with an edge if the edge is rejected by the Benjamini-Hochberg procedure and thus considered as a significant edge.

An R-GSN was constructed as follows:

1. Count the number of gene regulations where genes in GS1 regulate genes outside GS1 and the number of gene regulations where genes outside GS2 regulate genes in GS2.

2. Remove shared genes from GS1 and GS2 and count the number of gene regulations where the remaining genes in GS1 regulate the remaining genes in GS2.

3. Calculate the p-value by using the hypergeometric distribution using Formula (1), where N is the total number of gene regulations; n is the number of gene regulations where genes in GS1 regulate genes outside GS2; K is the number of gene regulations where genes in GS2 are regulated from genes outside GS2; and k is the number of gene regulations from genes in GS1 to GS2.

4. Adjust the p-value for multiple hypotheses to control the false discovery rate by using the Benjamini-Hochberg procedure with p-value ≤ 0.05.

5. Connect a pair of gene sets with a directed edge pointing from GS1 to GS2 if the edge is rejected by the Benjamini-Hochberg procedure and thus is significant.

R-GSNs were constructed separately for each gene set collection. Note that it is possible that R-GSNs contain a loop when both incoming and outgoing edges of a pair of gene sets are significant. R-GSNs are directed networks, while M-GSNs are undirected networks. Before comparing R-GSNs with M-GSN, the R-GSN was converted to an undirected network by discarding the directions of edges and removing loops.

### A disease specific gene set network

A disease specific gene set network was constructed in order to show how R-GSNs can help researchers explain disease complexity. Alzheimer's disease (AD) was chosen to be a case study. An AD gene list containing 347 genes from AlzGene database [17] was first collected. The five gene set collections were combined into a single global collection without removing and changing the original gene sets. From the list, AD related gene sets were selected from the global gene set collection. Then the AD specific gene set network was constructed. In the network, each node represents an AD related gene set. In order to find AD related gene set, the number of genes in each gene set found in the AD gene list was counted. Each AD gene list was treated as a new gene set. The same method used in constructing M-GSNs was used to calculate the p-value for each gene set. There were 216 gene sets which shared significantly high numbers of genes with the AD gene list. These AD related gene sets were collected and used to construct the AD specific R-GSNs.

### Network analysis

Three types of centrality [18] were calculated for each gene set in both R-GSNs and M-GSNs, *degree centrality, betweeness centrality*, and *closeness centrality*. igraph software package for R [19] was used in order to compute all network values. *Degree centrality *represents the number of edges upon a node. A gene set with a high degree centrality is likely to be an important gene set because it acts like a hub in the network. It can also be used for comparing two different types of networks. To conduct such comparison, we calculated Pearson's correlation coefficient of nodes' degree centrality between an M-GSN and an R-GSN. *Betweenness centrality *is defined as the number of times a node acts as a bridge along the shortest path between two other nodes. In a gene set network, a gene set with a high betweenness value is likely to be a part of several biological critical paths. *Closeness centrality *is defined as the inverse of the average length of the shortest paths between a node and all other nodes in a network. A node with a high closeness value is more central. In a gene set network, if a gene set with a high closeness value is disturbed, it is likely that a high number of gene sets will be affected by the gene set.

## Results and discussion

### Interpreting co-membership gene set network and regulatory gene set network

When a pair of gene sets in an M-GSN are connected, the interpretation depends on gene set data types. For pathway gene sets, an M-GSN represents pathway crosstalk; whereas for GO gene sets, an M-GSN represents protein moonlighting or gene sharing. When a pair of gene sets in an R-GSN are connected, they are connected by a directed edge. A directed edge in an R-GSN presents a possibility of one gene set regulating another.

According to Table 1, the numbers of unique genes from all gene set collections are not much different, where Reactome and GO Biological Process contain more genes than the others. The total count of unique genes from all collections is 11,111. We calculated the proportion of regulatory edges to nodes and normalized the proportion value by using number of unique genes. We used $\frac{r_{i}}{g_{i}/g}/s_{i}$, where *r_i _*is the number of gene is sets in collection *i; g_i _*is the number of unique genes in collection *i*; g is the total number of unique genes (11,111); and *s_i _*is the number of gene sets in collection *i*, to calculate the normalized proportion. In the R-GSN, GO Biological Process has the highest normalized proportion of edges to nodes (71.08) among the five gene set collections. These results indicated that pairs of biological process gene sets are more likely to have a regulatory relationship. In addition, according to the distribution of gene set sizes (Additional file 1), the five gene set collections have similar distributions. The distributions show that the five collections contain more small gene sets (size = 2-20) than large gene sets (size > 20).

**Table 1:** Summary of co-membership gene set networks and regulatory gene set networks for five gene set collections.

Collection	Number of gene sets	co-membership edges (M)	Regulatory edges (R)	Regulatory relationships	Shared edges ^a^	Number of genes	Normalized proportion of R edges to nodes
KEGG	186	2,230	4,452	3,274	1,461	5,267	50.49

Reactome	674	15,859	25,569	20,917	7,437	6,025	69.96

GO BP	825	33,055	32,607	27,513	10,354	6,178	71.08

GO CC	223	4,186	1,446	1,122	793	5,270	13.67

GO MF	396	3,178	2,620	2,404	503	5,314	13.83

^a^For regulatory edges, a pair of gene set can have a loop if both incoming and outgoing edges are significant. The number of regulatory edges is always greater than or equal to the number of regulatory relationships because one pair of gene sets either have or do not have regulatory relationship.

### Comparing the KEGG regulatory gene set network and the KEGG co-membership gene set network

In the M-GSN of KEGG, an edge between pathways can be considered as a pathway crosstalk [2,3]. Therefore, M-GSNs can be baselines to compare with R-GSNs. A regulatory relationship between pathways can provide complementary knowledge such as dysfunction of one pathway affecting function of other pathways. To investigate this knowledge, R-GSNs and M-GSNs of the KEGG pathways were compared.

The percentage of shared edges between M-GSNs and R-GSNs is the highest for KEGG Pathway (1461/(2230+3274-1461) = 36.14%). The low percentage of shared edges indicates that the R-GSN provides complementary information to the M-GSN. In addition, it is important to note that R-GSNs are constructed from gene regulations which were collected from high coverage data sources. Therefore, it is unlikely that R-GSNs depend on the quantity and the quality of experimental data.

Considering both the R-GSN and M-GSN of KEGG pathway gene sets, the most significant edge of the KEGG R-GSN is a regulatory relationship from "Cell cycle" to "Cytokine-cytokine receptor interaction" with p-value = 6.46E-75 (Table 2); whereas the co-membership edge between "Cell cycle" to "Cytokine-cytokine receptor interaction" has relatively low significance value ( 0.029). In addition, only 4 of the top 10 most significant regulatory edges are found in M-GSNs. These findings suggest that the R-GSN reveals additional knowledge to the M-GSN.

**Table 2:** Top 10 most significant regulatory edges in the KEGG regulatory gene set network.

Gene set 1 name	Gene set 2 name	P-value
Cell cycle	Cytokine-cytokine receptor interaction	6.46E-75

Cell cycle	Pathways in cancer	5.31E-55

Cell cycle	Toll-like receptor signaling pathway	1E-42

Cell cycle	Focal adhesion	2.22E-36

Cell cycle	Leishmania infection	2.63E-33

Hedgehog signaling pathway	Basal cell carcinoma	3.08E-33

p53 signaling pathway	Cytokine-cytokine receptor interaction	3.38E-33

RIG-I-like receptor signaling pathway	Toll-like receptor signaling pathway	3.39E-33

Cell cycle	Hematopoietic cell lineage	8.26E-33

Cell cycle	Jak-STAT signaling pathway	1.08E-31

The regulatory relationship is gene set 1 regulates gene set 2.

For the KEGG R-GSN, 7 of the 10 most significant regulatory edges are from the "Cell cycle" gene set to other 7 KEGG pathway gene sets (Table 2). This suggests that changing of "Cell cycle" pathway is likely to affect other pathways. This finding is corresponding to the fact that a cell cycle is a complex series of phenomena by which cellular material is duplicated and divided. Therefore if a cell cycle pathway does not function appropriately, other pathways such as Pathways in Cancer can be affected.

For the M-GSN, the KEGG pathway gene sets of Alzheimer's, Parkinson's, and Huntington's diseases have significant co-membership edges linking them together (Table 3). The three co-membership edges connecting the three neurodegenerative diseases are among the top 10 most significant co-membership edges suggesting that the three neurodegenerative diseases are highly related. In addition, 5 of the top 20 co-membership edges connect cancer related pathway gene sets. These 5 edges connect the "Pathways in cancer" gene set with 5 gene sets of cancers including "Small cell lung cancer", "Pancreatic cancer", "Melanoma", "Colorectal cancer", and Prostate cancer".

**Table 3:** Top 10 most significant co-membership edges in the KEGG co-membership gene set network.

Gene set 1 name	Gene set 2 name	P-value
Dilated cardiomyopathy	Hypertrophic cardiomyopathy (HCM)	2.9E-134

Oxidative phosphorylation	Parkinson's disease	5E-132

Huntington's disease	Parkinson's disease	2.2E-124

Alzheimer's disease	Parkinson's disease	8.9E-113

Drug metabolism - cytochrome P450	Metabolism of xenobiotics by cytochrome P450	2.5E-110

Alzheimer's disease	Huntington's disease	1.5E-106

Alzheimer's disease	Oxidative phosphorylation	1.5E-101

Huntington's disease	Oxidative phosphorylation	1.02E-96

Pathways in cancer	Small cell lung cancer	2.94E-91

Arrhythmogenic right ventricular cardiomyopathy (ARVC)	Hypertrophic cardiomyopathy (HCM)	1E-89

In addition, degree centrality (DC) of each node in both networks was calculated (Table 4). In the KEGG M-GSN, the "Pathways in cancer" gene set has the highest value of DC (0.39). In the KEGG R-GSN, the "Cell cycle" gene set has the highest DC (1.96) and the highest out-degree centrality (0.65). The gene set which has the highest in-degree centrality (0.41) is "Pathways in cancer". Using directionality information to calculate in-degree and out-degree centrality of the KEGG R-GSN, we can find the sink, "Pathways in cancer", and the source, "Cell cycle", gene sets.

**Table 4:** Top 5 degree centrality pathway of KEGG co-membership gene set network (left) and regulatory gene set network (right).

KEGG co-membership network		KEGG regulatory network	
**Name**	**DC**	**Name**	**DC**

Pathways in cancer	0.39	Cell cycle	1.96

MAPK signaling pathway	0.36	T cell receptor signaling pathway	1.55

T cell receptor signaling pathway	0.36	Chemokine signaling pathway	1.49

Chemokine signaling pathway	0.36	ErbB signaling pathway	1.48

Natural killer cell mediated cytotoxicity	0.36	p53 signaling pathway	1.48

**Figure 1:**
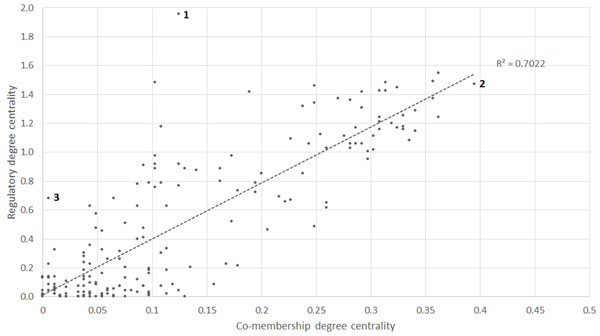
**Correlation between DC of the KEGG M-GSN and DC of the KEGG R-GSN**. Pathway number 1 is Cell cycle; pathway number 2 is Pathways in cancer; and pathway number 3 is Maturity onset diabetes of the young.

After the DC value for each gene set was calculated, the correlation between DC of the KEGG M-GSN and DC of the KEGG R-GSN was calculated. The correlation coefficient is 0.84 and R-squared value is 0.70 (Figure 1). This result suggests that the gene set which is important in the M-GSN is likely to be important in the R-GSN and vice versa. We found three interesting outliers. Pathway number 1, which has regulatory DC = 1.96 and co-membership DC = 0.12, is "Cell cycle" suggesting that the Cell cycle pathway does not tend to share genes, but to have regulatory relationship with other pathways. Pathway number 2, which has regulatory DC = 1.47 and co-membership DC = 0.39, is "Pathways in cancer" suggesting that Pathways in cancer shared high number of genes with several pathways and their genes also regulate the unique genes of other pathways. Pathway number 3, which has regulatory DC = 0.68 and co-membership DC = 0.005, is "Maturity onset diabetes of the young". This pathway shares 6 genes with only "Type II diabetes mellitus pathway".

**Figure 2:**
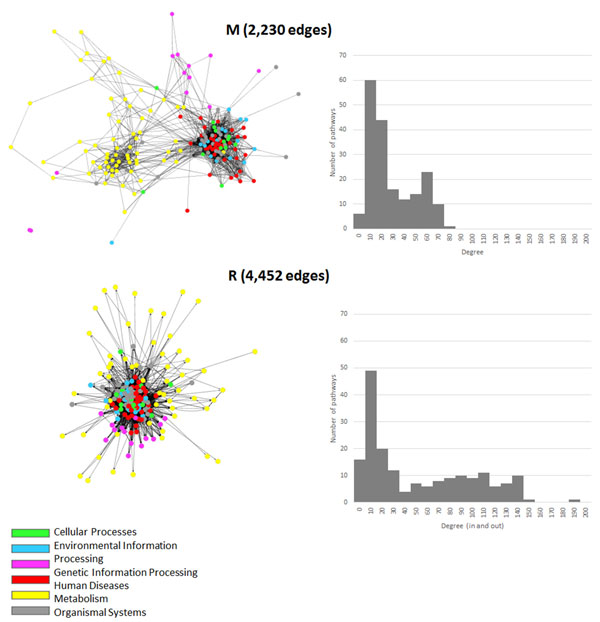
**Gene set networks of KEGG gene sets**. (M) is the KEGG M-GSN and (R) is the KEGG R-GSN. Node colors represent different classes of pathways. The networks were drawn by Cytoscape using Cytoscape's BioLayout.

While the correlation between DC of the KEGG M-GSN and DC of the KEGG R-GSN is as high as 0.84, the topologies of the networks are different (Figure 2). This suggests that the two networks can be used to explain different phenomenon in biological systems.

### Constructing an exclusive R-GSN

**Figure 3:**
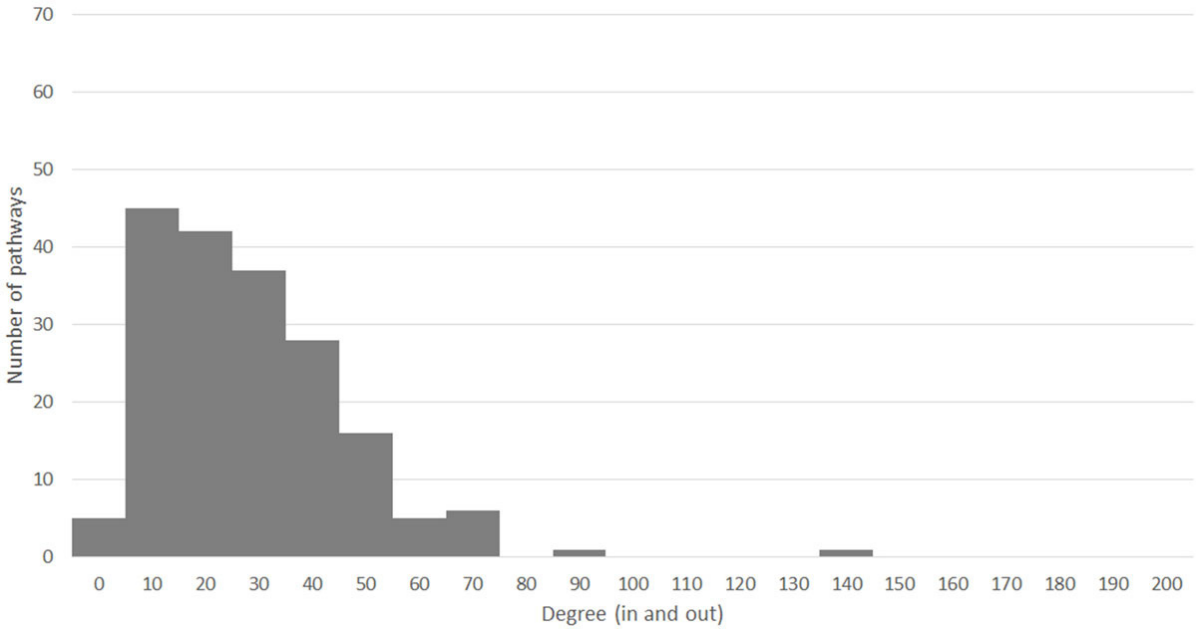
**Degree distribution of KEGG exclusive R-GSN**. This figure show the degree distribution of KEGG exclusive regulatory gene set network. Because the regulatory gene set network is a directed network, the degree of each nodes was counted by summing in-degree and out-degree.

We constructed a KEGG exclusive R-GSN (R-GSN minus M-GSN) that contains only exclusive edges in the R-GSN (Figure 3). The correlation of DC between the KEGG exclusive R-GSN and the KEGG R-GSN is 0.81. The correlation of DC between the KEGG exclusive R-GSN and the KEGG M-GSN is 0.44, while the correlation of DC between the KEGG non-exclusive R-GSN and the KEGG M-GSN is 0.84. These suggest that the exclusive R-GSN can reveal important gene sets that are not likely to be revealed by M-GSN.

The directionality information from a regulatory gene set network revealed "sink" and "source" gene sets in addition to "hub" gene sets, which can be revealed by constructing. Table 5 shows the top 10 highest out-degree centrality gene sets (right) and top 10 highest in-degree centrality gene sets (left) of KEGG exclusive R-GSN. "Cell cycle", "p53 signaling pathway", and "TGF-beta signalling pathway" are among the top 3 highest out-degree centrality which can be the sources. The top 3 highest in-degree centrality gene sets, "Cell cycle", "Hematopoietic cell lineage", and "Cytokine-cytokine receptor interaction", are the sinks.

**Table 5:** Top 10 highest out-degree centrality and In-degree centrality of the KEGG exclusive R-GSN.

Name	DC (out)	Name	DC (in)
Cell cycle	0.54	Cell cycle	0.24

p53 signaling pathway	0.44	Hematopoietic cell lineage	0.21

TGF-beta signaling pathway	0.37	Cytokine-cytokine receptor interaction	0.20

Bladder cancer	0.32	Systemic lupus erythematosus	0.17

Small cell lung cancer	0.25	Leishmania infection	0.17

Chronic myeloid leukemia	0.24	Basal cell carcinoma	0.17

Jak-STAT signaling pathway	0.24	Cell adhesion molecules (CAMs)	0.17

Huntington's disease	0.24	Graft-versus-host disease	0.17

Non-small cell lung cancer	0.23	Viral myocarditis	0.16

Wnt signaling pathway	0.22	p53 signaling pathway	0.15

### Comparison of the KEGG co-enrichment network and the KEGG regulatory network

In the previous analysis, we used the KEGG M-GSN as a baseline and compared it with the KEGG R-GSN. Several relationships between gene sets were found exclusively in the R-GSN. In order to validate both the KEGG M-GSN and the KEGG R-GSN, the two GSNs were compared with the KEGG co-enrichment network (E-GSN) obtained from the study of Jignesh, et al. [11]. To construct an E-GSN, they integrate experimental gene lists and link two gene sets if the unique genes of the two gene sets are consistently enriched together across many experimentally derived gene lists. Therefore, edges found in the KEGG R-GSN should also be found in the KEGG E-GSN.

**Figure 4:**
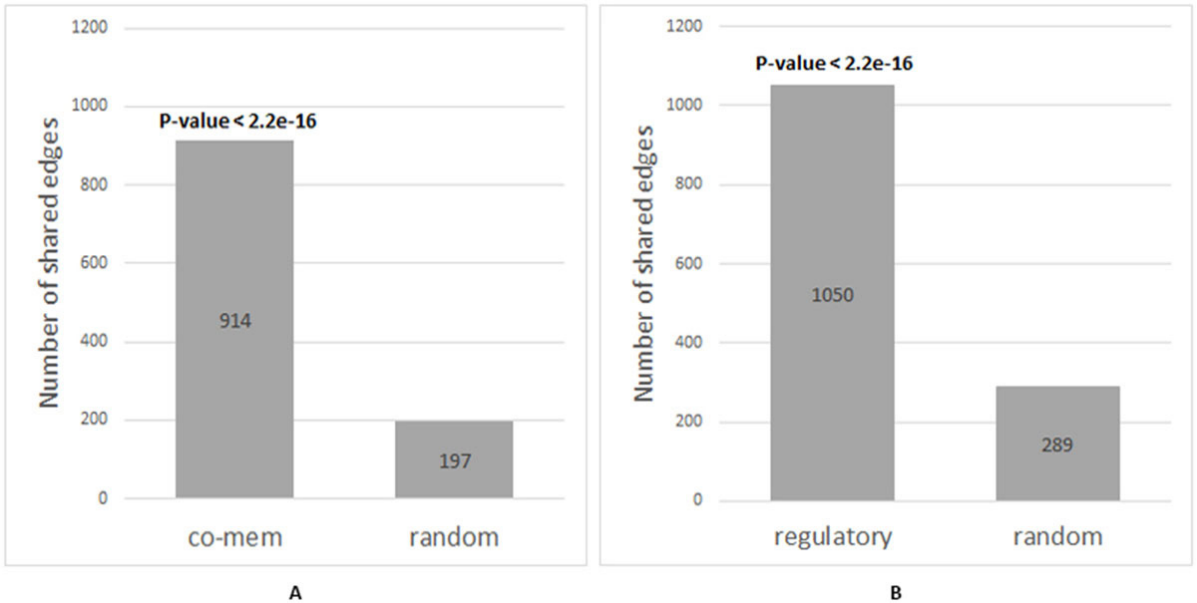
**Number of shared edges between the KEGG M-GSN and the KEGG E-GSN and between the KEGG R-GSN and the KEGG E-GSN**. (A) A graph showing number of edges in the KEGG E-GSN that shared with the KEGG M-GSN and shared with 1,000 random networks. For the number of shared edges between the 1,000 random network and the KEGG E-GSN, the average is 197; the minimum is 162; and the maximum is 236. (B) A graph showing number of edges in the KEGG E-GSN that shared with the KEGG R-GSN and shared with 1,000 random networks. For the number of shared edges between the 1,000 random networks and the KEGG E-GSN, the average is 289; the minimum is 240; and the maximum is 333.

The total number of edges found in both the E-GSN and the R-GSN is 1,050, which accounts for 67.48% of the total number of edges in the E-GSN. The total number of edges found in both the E-GSN and M-GSN is 914, which is equal to 58.74% of the total number of edges in the E-GSN. In order to calculate the significance value of the number of shared edges, the KEGG E-GSN was compared with random networks. We randomly generated 1,000 networks using all the 187 gene sets from KEGG. To calculate the significance value of the number of shared edges between the KEGG E-GSN and the KEGG M-GSN, each of the 1,000 random networks contains 2,230 edges, which are equal to the number of edges found in the KEGG M-GSN. Then Fisher's exact test was used for calculating the p-value for the number of shared edges. The p-value is < 2.2e-16 (Figure 4A). To calculate the significance value of the number of shared edges between the KEGG E-GSN and the converted KEGG R-GSN, each of the 1,000 random networks contains 3,274 edges. Then Fisher's exact test was used for calculating the p-value for the number of shared edges. The p-value is < 2.2e-16 (Figure 4B).

The number of shared edges between the KEGG E-GSN and the KEGG R-GSN is significantly high. This is corresponding to the fact that a pair of gene sets with strong regulatory relationship should be connected with a co-enrichment edge. The number of shared edges between the KEGG E-GSN and the KEGG M-GSN is also significantly high. This is also corresponding to the fact that a pair of gene sets with a high number of shared genes should be connected with a co-enrichment edge.

### A disease specific regulatory gene set network

**Figure 5:**
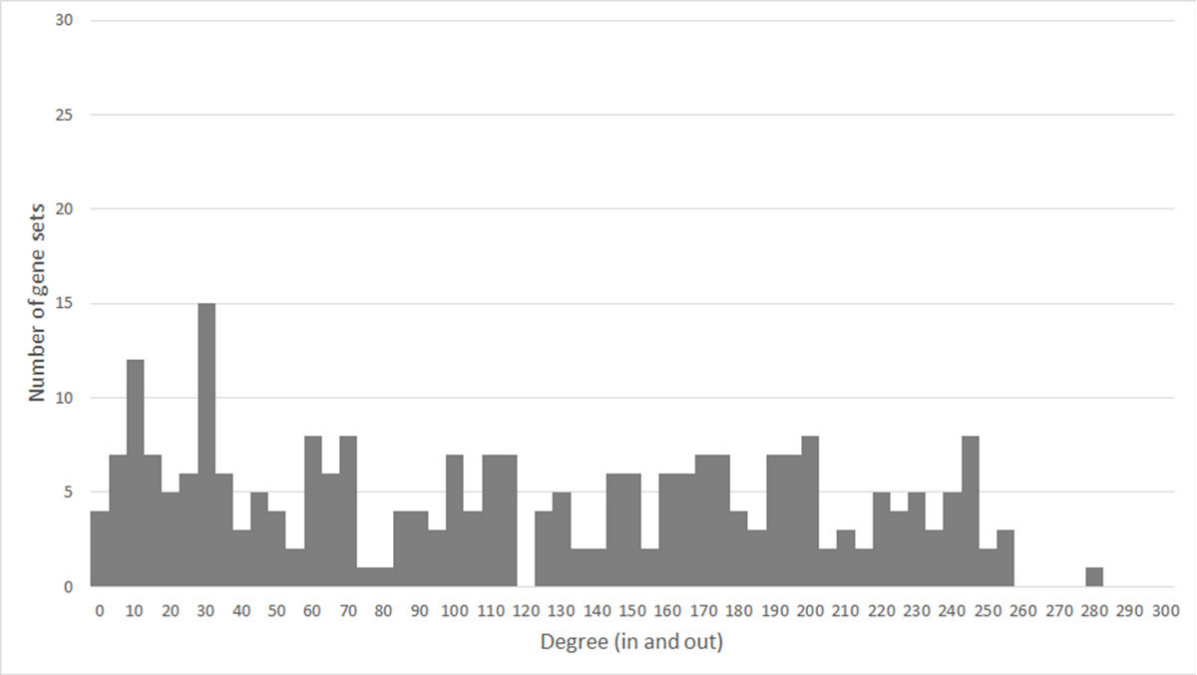
**Degree distribution of the AD specific R-GSN**. Degree distribution of the AD specific R-GSN containing 261 gene sets and 15,178 regulatory edges.

**Figure 6:**
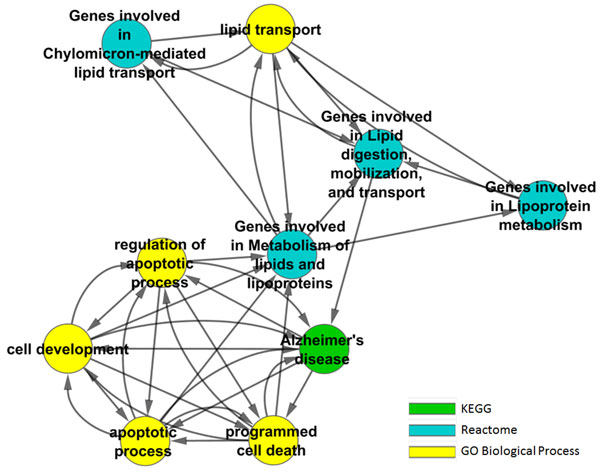
**A R-GSN of the top 10 AD related gene sets**. A regulatory gene set network of the top 10 AD related gene sets. Node colors represent different collections of gene sets. The networks were drawn by Cytoscape using Cytoscape's BioLayout.

The number of shared genes between the AD gene list (Additional file 2) and each gene set in the global gene set collection was counted. Out of the 2,314 gene sets, 261 have significant number of shared genes. Among the 261 AD gene sets, 42 are from KEGG; 59 are from Reactome; 37 are from GO Molecular Function; 105 are from Go Biological Process; and 18 are from GO Cellular component. Figure 5 shows the degree distribution of AD-specific R-GSN constructed based on these 261 AD gene sets. In addition, Table 6 shows the top 10 AD related gene sets arranged by the significant values. "Genes involved in Lipid digestion, mobilization, and transport" gene set has the highest significant value, 2.61E-13. The significant value was calculated by using Formula (1) where there are 46 genes and 16 genes were found in the AD gene list. The AD related R-GSN of the top 10 gene sets are presented in Figure 6.

**Table 6:** Top 10 AD related gene sets arranged by p-value.

Gene set name	Collection	Size	AD genes	P-value
Genes involved in Lipid digestion, mobilization, and transport	Reactome	46	16	2.60794E-13

Alzheimer's disease	KEGG	169	28	3.33483E-13

lipid transport	GO Biological Process	28	13	5.6133E-13

Genes involved in Lipoprotein metabolism	Reactome	28	13	5.6133E-13

apoptotic process	GO Biological Process	431	42	3.80595E-11

programmed cell death	GO Biological Process	432	42	4.09448E-11

Genes involved in Metabolism of lipids and lipoproteins	Reactome	478	44	8.10901E-11

Genes involved in Chylomicron-mediated lipid transport	Reactome	16	9	2.51488E-10

cell development	GO Biological Process	577	48	3.3663E-10

regulation of apoptotic process	GO Biological Process	341	34	1.66543E-09

We then investigated the top 10 degree centrality gene sets. Table 7 shows that "signal transduction" from GO biological process has the highest value of DC suggesting that the signal transduction process is very important in AD. Searching on PubMed found more than 2,000 publications discussing the relationship between signal transduction abnormality and Alzheimer's disease.

**Table 7:** Top 10 degree centrality gne sets among 261 AD related gene sets.

Name	Collection	DC	in-DC	out-DC
signal transduction	GO biological process	1.07	0.62	0.45

Cytokine-cytokine receptor interaction	KEGG	0.97	0.53	0.44

intracellular signal transduction	GO biological process	0.97	0.47	0.49

protein metabolic process	GO biological process	0.96	0.43	0.54

T cell receptor signaling pathway	KEGG	0.95	0.44	0.51

cellular protein metabolic process	GO biological process	0.94	0.40	0.54

receptor binding	GO molecular function	0.94	0.44	0.49

cellular macromolecule metabolic process	GO biological process	0.94	0.40	0.54

apoptotic process	GO biological process	0.93	0.44	0.49

programmed cell death	GO biological process	0.93	0.44	0.49

Considering the directionality of the AD specific R-GSN, "signal transduction" has the highest in-DC (0.62). Gene sets which have the highest out-DC (0.54) are "cellular protein metabolic process" and "cellular macromolecule metabolic process" (Additional file 3). These results show that directionality information from the R-GSN enables us to identify, in AD context, a sink gene set, "signal transduction", and source gene sets, "cellular protein metabolic process" and "cellular macromolecule metabolic process".

Furthermore, the closeness and betweenness of each gene set in the AD specific R-GSN were computed. Note that the directionality of each edge in the network was considered when we calculated the shortest path for the network in order to calculate the closeness and the betweenness. For the closeness centrality, "signal transduction" from the GO biological process, "Pathways in cancer" from KEGG, and "protein metabolic process" from the GO biological process have the highest closeness values (0.190, 0.188, and 0.188, respectively). These results suggest that inappropriate functions of the three gene sets are likely to affect high number of gene sets in AD. For the betweenness centrality, "Pathways in cancer" from KEGG, "system development" from GO biological process, and "Leishmania infection" from KEGG have the highest betweenness values (1,225.04, 1168.99, and 1146.79, respectively). These results suggest that the three gene sets are likely to be on the critical path of biological functioning for AD patients.

## Conclusions

Co-membership gene set networks (M-GSNs) and regulatory gene set networks (R-GSNs) for the five different gene set collections were constructed, compared, and studied for their biological relevance in this study. The results show that new R-GSNs can provide complementary biological information to conventional M-GSNs. The results also show that while the correlation between the degree centrality of the KEGG M-GSN and the degree centrality of the KEGG R-GSN is relatively high, the topologies of the networks are totally different. This suggests that the two networks can be employed to explain different phenomenon in biological systems.

Validating KEGG M-GSNs and KEGG R-GSNs separately against the KEGG co-enrichment networks (E-GSNs) [11] reveals that the numbers of shared edges are significantly high. The results suggest that the use of E-GSN to validate M-GSN and R-GSN could be a better way to describe experiments. Moreover, the R-GSN specific to Alzheimer's disease shows that the network can be used as a good high-level mechanistic model complementary to a more difficult-to-derive gene regulatory network model towards a systematic understanding of the disease mechanism.

In this study, we describe methods for constructing both global and disease specific R-GSNs. They enable future research on systems biology and translational bioinformatics. Since the underlying gene regulation data are collected from high quality and high coverage data sources, the directed edges in the R-GSN do not tend to depend on the number and the quality of experimental data. Moreover, directionality information from the R-GSN enables the finding of source gene sets and sink gene sets, which might be important for drug discovery or drug repositioning.

Tissue-specific and disease-specific gene regulations were not used in this study. Therefore, the GSNs obtained might be generally applicable but should be carefully reviewed and curated when they are to be used in specific disease contexts where differential gene regulations exist extensively. However, this limitation can be alleviated by constructing only condition-specific GSNs instead of global GSNs in the future. The framework in this study can also be later extended by collecting higher resolution such as tissue specific and disease specific gene regulation data and gene set data. Separately, we have been developing a comprehensive database populated with new gene sets and all relationships identified or integrated between genes and gene sets into an online resource called PAGER [20] to put the method developed in this work into a web application for biological users to explore.

## List of abbreviations used

GSN: Gene set network; M-GSN: Co-membership gene set network; E-GSN: Co-enrichment gene set network; L-GSN: Linkage gene set network; R-GSN: Regulatory gene set network; DC: Degree centrality; M: Co-membership edge; E: Co-enrichment edge; R: Regulatory edge; L: Linkage edge.

## Competing interests

The authors declare that they have no competing interests.

## Authors' contributions

Jake Y Chen conceived of this work, guided the research team by providing ideas and feedback along the way, and revised the manuscript. Chayaporn collected the data, constructed gene set networks, analyzed the networks, and wrote the manuscript. Liugen assisted network visualization and revised the manuscript.

## Supplementary Material

Additional File 1 **Distribution of the sizes of gene sets in each collection**. This file contains five histograms presenting distribution of the sizes of gene sets in each of the five gene set collections. Click here for file

Additional File 2 **AD gene list**. This file contains a list of AD related gens. Click here for file

Additional File 3 **Centrality values of each gene set in the AD specific gene set network**. This file contains centrality values, including degree centrality, closeness centrality, and betweenness centrality, of 261 AD related gene sets in the AD specific R-GSN. Click here for file

## Declarations

Publication costs for this article were funded by the corresponding author's affiliated institutions, Indiana University School of Informatics and Computing in the United States and Wenzhou Medical University in China.

This article has been published as part of *BMC Genomics *Volume 16 Supplement 11, 2015: Selected articles from the Fourth IEEE International Conference on Computational Advances in Bio and medical Sciences (ICCABS 2014): Genomics. The full contents of the supplement are available online at http://www.biomedcentral.com/bmcgenomics/supplements/16/S11.
